# The Relationship Between Resilience and the Intention to Leave Among Staff Nurses at Governmental Hospitals in the Al-Baha Region of Saudi Arabia

**DOI:** 10.7759/cureus.56699

**Published:** 2024-03-22

**Authors:** Sara Al-shomrani, Sabah M Mahran, Ohood Felemban

**Affiliations:** 1 Nursing, King Abdulaziz University, Jeddah, SAU; 2 Public Health/Nursing Administration, College of Nursing, King Abdulaziz University, Jeddah, SAU; 3 Public Health/Community and Primary Healthcare, King Abdulaziz University, Jeddah, SAU

**Keywords:** hospitals, turnover intention, intention to leave, staff nurse, resilience

## Abstract

Background

Nurses with high intent to leave can cause substantial problems for healthcare organizations, such as staffing shortages and higher expenses due to hiring and onboarding new nurses. In light of the increasing demands placed on nurses in understaffed and overloaded healthcare systems, nurses frequently face various pressures and difficulties in their field of work, including high workloads, irregular hours, complicated patients, and infectious disease exposure; resilience is critical for handling stress and hardship at work. Nurses will thus retain their jobs for longer. This study aimed to determine the relationship between resilience and the intention to leave among staff nurses.

Methods

This study utilized a quantitative, cross-sectional correlation design. It comprised three Saudi Ministry of Health-affiliated facilities in the Al-Baha region (King Fahad Hospital, Prince Mashari Hospital, and Mikhwah General Hospital). The study sample comprised nurses employed in critical areas and inpatient and outpatient hospital departments using convenience sampling and inclusion and exclusion criteria. An online questionnaire involving three sections was given out. The first part collected sociodemographic data, the second part included the Connor-Davidson Resilience Scale 25 (CD-RISC-25), and the third included the Anticipated Turnover Scale (ATS).

Results

This study found a moderate degree of intention to leave and resilience. Most participants in the survey held a bachelor's degree (75.8%), and around 87.1% of the sample consisted of women. About half of the sample (57.2%) were married; 67.6% of the participants were not Saudi nationals; and regarding the number of children, the majority (53.8%) were childless. Overall, 318 nurses working in acute and outpatient departments and critical regions participated. According to the study, 73.3% of the participants reported a moderate intention to quit, whereas 50.9% had moderate resilience. Similarly, a significant negative relationship was found between nurses' intention to leave and resilience.

Conclusions

In the current study, resilience has a statistically significant negative relationship with the nurses’ intention to leave. Hospital management must consider the amount of work and the excessive work schedule to reduce nurses’ intentions to leave. One way to do this is by assigning tasks to employees, minimizing their workload through flexible work schedules and shorter duty hours, and fostering teamwork among coworkers by ensuring clear communication and cooperation. Interventions like orientation programs for new nurses, regular meetings, seminars, and training sessions can improve nurse resilience.

## Introduction

Nurses are the primary providers of patient care, and they shape the direction of the healthcare system. As with other healthcare professionals, nurses often face a range of stressors and challenges in their work environment, such as heavy workloads, long hours, complex patients, and exposure to infectious diseases. Albougami et al. [1] conducted a cross-sectional study in Saudi Arabia using a sample of 318 staff nurses to examine the factors that influence the intention of Saudi Arabian nurses to leave their current positions. They concluded that one of the significant issues many healthcare organizations face is employee turnover, leading to a shortage of nurses and job stress due to increased workloads. Therefore, higher resilience among nurses has been associated with better outcomes regarding their physical and mental health. Moreover, they are less likely to experience turnover intentions, burnout, or job dissatisfaction. In response to the increasing demands placed on nurses working in overloaded and inadequate healthcare systems, developing personal resilience is essential for overcoming the stress and hardship associated with their working area [2].

Indeed, the concept of resiliency in nursing has become increasingly important. In numerous studies, the relationships among resilience and nurse turnover intentions, job satisfaction, and job stress have been extensively discussed in the literature. Hudgins [3] conducted a quantitative study with a sample of 89 nurse leaders in southwest Virginia to determine the relationships among resilience, job satisfaction, and anticipated turnover among nurse leaders. Hudgins further found significant relationships among resilience, job satisfaction, and anticipated turnover. A different study conducted in China demonstrated a serial mediation model in which nurses’ intent to stay was enhanced by positive resilience, which first encouraged perceived professional advantages and then increased posttraumatic growth [4]. Therefore, nurse leaders greatly help with developing a resilient nursing workforce. To prevent burnout, perform better patient care, and preserve the financial stability of healthcare institutions, it is critical to support nurses’ resilience [5]. In this light, the current study sought to investigate the role of resilience in predicting nurses’ intentions to leave.

Nurse intention to leave is acknowledged not just because of their maladjustment to their clinical or employment settings but also because it is an organizational issue. Saudi Arabia’s rapidly growing population necessitates developing and maintaining a stable nursing workforce. In addition, nurses’ intention to leave has a negative impact on the patients’ need for appropriate, high-quality care. Moreover, a large amount of research has shown high turnover increases the cost of hiring and replacing staff at the organizational level. They have reduced productivity and job satisfaction and decreased organizational performance [1]. However, because of the increasing expatriate worker turnover and the minimal recruitment of Saudi residents, there is a severe shortage of competent and experienced nurses [6]. Saudi Arabia has a severe nursing shortage and relies on expatriate nurses. According to statistics from 2018, there were 184,565 nurses in the kingdom, but only 70,319 (or around 38%) were Saudi citizens. Another statistic from 2021 is that approximately 43% of Saudi citizens and 57% of non-Saudi nurses are foreigners. This presents unique challenges for the country [7,8].

A study conducted in Lebanon by Alameddine et al. [9] highlighted the significance of personal resilience in lowering turnover during crises and discovered a strong negative association between nurses’ resilience and the intention to resign. Moreover, Coa and Chen’s [10] descriptive cross-sectional study in China investigated the moderating roles of compassion fatigue and work engagement on the connection between turnover intention and resilience in dialysis nurses. Through improved work engagement and decreased compassion fatigue in dialysis nurses, they discovered greater resilience lowers intentions to leave the profession. There are few studies on Saudi Arabian nurses’ resilience and intention to leave. Due to this, a knowledge gap exists about how these two variables are related. Therefore, this study will contribute to the development of the nursing profession, including nursing practice and organizational performance. Moreover, it could provide insight that might help reduce the nurses’ intention to leave Saudi healthcare organizations.

## Materials and methods

Aim and design

This quantitative cross-sectional correlation study aimed to determine the relationship between staff nurses' intention to leave and resilience, assess the level of personal resilience, and identify the level of staff nurses' intention to leave.

Setting and sample

The nurses working in outpatient clinics, critical care units, emergency rooms, medical departments, obstetrics and gynecology departments, pediatric departments, and surgical departments at three hospitals in the Al-Baha region of Saudi Arabia comprised the study population. Based on my search through the database, I chose the three hospitals in the Al-Baha region mainly because more research needs to be conducted there. The nursing field needs to be researched in Al-Baha because nurses experience high stress due to overload, which leads to a high turnover rate. During the study, convenience sampling was used to ensure surveillance of all staff nurses working in Al-Baha hospitals' inpatient and outpatient areas. To be included in the study, the nurses who took part in it had to be (a) aged 30 to 50 years old with experience from one to 10 years and more; (b) registered nurses working in the clinical areas of the inpatient and outpatient units; (c) registered and certified nurses; and (d) full-time employees of the organization being studied. Nurse administrators, managers, interns, and students were excluded. They are less involved in the clinical situation and may experience different challenges, which could affect the study results.

A sufficient sample size is required to answer the research question because a smaller sample size could impair the dependability of results. Overall, 851 clinical nurses worked in the studied organizations' inpatient and outpatient units. The sample size for this study was calculated using the Raosoft electronic online website, which automatically calculates the appropriate sample size with a 5% margin error and a 95% confidence interval. Considering lost or unusable data and dropping rates, another 20% of respondents were accounted for in the study's total sample. Hence, the recommended sample size for this study to establish representative results is 318 participants.

Data collection and instruments

Once the researcher has acquired approval from all study parties, the researcher visited the hospital to meet with the chief nursing officer (CNO) for each institution. The researcher briefly summarized the purpose of the study, which was to determine the relationship between resilience and nurses' intention to leave. All staff members who meet the inclusion requirements (i.e., registered nurses working in the inpatient and outpatient units' clinical departments) were emailed and provided the link by the CNO. Two weeks later, the participants received a reminder from the CNO. Due to the participants' initially weak replies, the researcher had to make multiple hospital visits to encourage them to complete the survey and prolong it for two months to receive responses from more nurses. The link to the questionnaire was accessible until enough samples were collected (318), and the study's data collection period lasted from February 15 to April 30. Data were collected using self-administered online questionnaires, which consisted of three parts: (1) demographic and working characteristics, (2) the Connor-Davidson Resilience Scale 25 (CD-RISC-25), and (3) the Anticipated Turnover Scale (ATS).

Demographic and Working Characteristics

The researcher created the questionnaire’s section on sociodemographic data, and it collected the following: age, gender, marital status, number of children, nationality, education level, years of nursing experience, hospital where the nurse worked, unit or department where the nurse worked, working shift, monthly salary, and hours worked each day.

Connor-Davidson Resilience Scale 25 (CD-RISC-25)

The first tool was taken from Connor-Davidson [11] and was employed to gather data regarding resilience. It includes 25 items in total. A five-point Likert scale ranging from 0 to 4 was as follows: 0 = not true at all, 1 = rarely true, 2 = sometimes true, 3 = often true, and finally, 4 = true nearly all of the time. These ratings produced scores ranging from 0 to 100, with a response rate of less than 50 being weak, from 50 to 75 being average, and more than 75 having higher values, suggesting better nurse resiliency.

Anticipated Turnover Scale (ATS)

Nurses' intentions to leave their current positions voluntarily - either through internal or external turnover or by ending their nursing careers - were investigated using the second tool, the ATS (as adopted by Hinshaw and Atwood [12]). The ATS is a 12-item, seven-point Likert scale with responses ranging from 1 to 7, from strongly agreeing (7) to strongly disagreeing (1). A higher ATS score suggests that nurses are more likely to leave the organization. Wherever else, a lower score indicates nurses are less likely to leave their organization. The range of scores for the ATS instrument was between 12 and 84, with a response rate of less than 50 being weak, from 50 to 75 being average, and more than 75 having a high intention to leave. Items 2, 4, 5, 7, 11, and 12 were positive, whereas items 1, 3, 6, 8, 9, and 10 were negative. If the item was positive, it was graded on a scale of 7 to 1, with AS equaling seven points and DS equaling one. A negative survey item was graded on a scale of 1 to 7, with AS equaling one point and DS equaling seven. A score of 1 indicates that one is not planning to leave one’s nursing job, whereas a score of 7 indicates that one is planning to leave one’s nursing job. For questions about the study, the researcher offered a phone number, email address, and communication channels. 

Validity and reliability of the tools

A group of associate professors and expert assistants from King Abdul-Aziz University's nursing faculty evaluated the questionnaire. Before releasing the questionnaire to participants, experts with experience and understanding of the study issue examined it to ensure the items were valid, consistent, and easy to comprehend. According to the jury, the assertions in the questionnaire were reasonable, consistent, and relevant to the study's objective of gathering information and evaluating the questionnaire's applicability. It was concluded that no adjustments were required. The standard questionnaire was translated into Arabic to make it more understandable for nurses who do not know English. After completing a pilot survey, 31 nurses (10% of the sample) were questioned regarding the questionnaire's readability, relevance, design, clarity, and time required. A questionnaire's validity is determined by how well it reflects the concept under study. For reliability, the Cronbach's alpha coefficient was outstanding (0.90) for resilience, and the anticipated turnover scale was excellent (0.81).

Data analysis

The data were extracted to an Excel sheet. To maintain participant confidentiality, the names were encoded with numbers. Therefore, the data were changed to be used with IBM SPSS Statistics for Windows, version 23 (released 2015; IBM Corp., Armonk, New York, United States). A chi-square test was employed in relation to the staff nurses' resilience. Using a one-sample *t*-test, the total mean score for all categories was determined for the anticipated turnover among nursing staff. In addition, the relationship between demographic data, the overall resilience score, and the nurses' intention to leave was examined using an independent sample *t*-test and a one-way ANOVA. Moreover, the Pearson correlation test evaluated the relationship between the intention to leave and the resilience of staff nurses. An association or connection is deemed significant if the *p*-value is less than 0.05. The researcher used tables and graphs to help with data analysis.

Ethical considerations

Official approval was obtained from the Directorate of Health Affairs in the Al-Baha region of Saudi Arabia (IRB-23-08-2022/1), and the Research Ethics Committee of the King Abdulaziz University Faculty of Nursing provided official approval (Ref. No. 2M. 47). The study's goal, potential dangers, and the option to withdraw at any time if one did not participate were explained to the CNO and nurses who met the selection criteria before data collection. The investigators adhered to ethical principles, such as privacy, secrecy, autonomy, and self-determination. By completing the questionnaire, the participants were considered to have consented to participate in the study. To prevent bias during the data collection, the research team ensured they knew no participants. In addition, the data were stored in a secure location; only the research team could access the data, and after the study is finished, the data gathered from the questionnaire will be appropriately destroyed.

## Results

Demographic characteristics

Table 1 reveals that 150 participants (47.2%) fell within the 30-40-year range, whereas 49 (15.4%) were between 40 and 50. In addition, more than three-quarters of the sample studied (277, 87.1%) were female, with men accounting for the remaining 41 (12.9%). Regarding educational attainment, bachelors (241, or 75.8%) outnumbered diploma holders (49 or 15.4%). Approximately half (182, 57.2%) of the studied sample were married, whereas 122 (38.4%) were single. Non-Saudis comprised the majority of participants (215, 67.6%), whereas Saudis comprised 103 (32.4%). A small majority of the respondents (171, 53.8%) had no children. About the working hospital, 117 (36.8%) of the participants were affiliated with King Fahad Hospital, 109 (34.3%) with Al-Mikhwah General Hospital, and 92 (28.9%) with Prince Mashari Hospital. Examining years of experience at the current hospital, 132 (41.5%) had one to five years of experience. A smaller segment (38, 11.9%) had less than a year's experience. Finally, regarding monthly allowances, 174 (54.7%) earn less than 10,000 SR.

**Table 1:** Demographic characteristic data in the study sample (n = 318).

Demographic characteristics	N	%
Gender		
Female	277	87.1
Male	41	12.9
Nationality		
Saudi	103	32.4
Non-Saudi	215	67.6
Age		
<30 years	110	34.6
>30-40years	150	47.2
>40-50years	49	15.4
≥50 years	9	2.8
Marital status		
Married	182	57.2
Single	122	38.4
Widowed	4	1.3
Divorced	10	3.1
Number of children		
No. of children	171	53.8
1 child	51	16.0
2 children	57	17.9
3 and more children	39	12.3
Education		
Diploma	49	15.4
Bachelor's degree	241	75.8
Master's degree	24	7.5
Doctoral degree	4	1.3
Working hospital		
King Fahad Hospital	117	36.8
Prince Mashari Hospital	92	28.9
Al-Mikhwah general Hospital	109	34.3
Years of experience at the current hospital		
<1 year	38	11.9
>1-5 years	132	41.5
>5-10 years	73	23.0
>10 years	75	23.6
Monthly allowance		
<10 SR	174	54.7
10-20 SR	95	29.9
>20 SR	49	15.4

Table 2 provides a comprehensive overview of the working characteristics of the studied sample. Regarding working departments, most participants worked in other units (56, 17.6%), whereas 51 worked in outpatients (16.0%). Still, the pediatric departments were 41 (12.9%). Regarding working shifts, most participants rotated day, evening, and night (186, 58.5%); finally, regarding working time (hours/day), most participants worked 12 hours (145, 45.6%).

**Table 2:** Distribution of the working characteristics of staff nurses (n = 318).

Working characteristics	N	%
Working departments		
Critical care units	33	10.4
Surgical departments	38	11.9
Obstetrics and gynecology	29	9.1
Emergency department	37	11.6
Medical departments	33	10.4
Outpatients	51	16.0
Pediatric departments	41	12.9
Other	56	17.6
Working shifts		
Day	119	37.4
Evening	13	4.1
Rotating day, evening, and night	186	58.5
Working time (hours/day)		
Eight hours	130	40.9
Ten hours	43	13.5
Twelve hours	145	45.6

Table 3 shows the frequency and percentage of resilience using the Connor-Davidson Resilience Scale 25 (CD-RISC-25) employed in the study sample (n = 318), whereas the highest item was "when there are no clear solutions to my problems, sometimes fate or God can help” (Item 3) with 81.05%. By contrast, the lowest item was “I can make unpopular or difficult decisions that affect other people if it is necessary” (Item 18) with 59.43%. Furthermore, the results found that all items have a significant relationship with resilience (*p*-value < 0.05), as represented in Table 3.

**Table 3:** Frequency and percentage of resilience using the Connor-Davidson Resilience Scale 25 (CD-RISC-25) employed in the study sample (n = 318). The *p*-value has been calculated using the Chi-square test. * Significant at the *p *< 0.05 level.

Items		CD-RISC-25					%	Chi-square	
		Not true at all	Rarely true	Sometimes true	Often true	True nearly all the time		X^2^	P-value
Adapt when changes occur.	N	7	20	121	102	68	66.04	155.553	0.000*
	%	2.2%	6.3%	38.1%	32.1%	21.4%			
Having a secure relationship.	N	21	34	94	107	62	62.19	86.497	0.000*
	%	6.6%	10.7%	29.6%	33.6%	19.5%			
Sometimes fate or God can help.	N	6	14	48	79	171	81.05	279.767	0.000*
	%	1.9%	4.4%	15.1%	24.8%	53.8%			
Deal with whatever comes my way.	N	4	19	99	118	78	69.42	156.623	0.000*
	%	1.3%	6.0%	31.1%	37.1%	24.5%			
Dealing with new challenges and difficulties.	N	5	11	70	116	116	75.71	184.484	0.000*
	%	1.6%	3.5%	22.0%	36.5%	36.5%			
See the humorous side of things.	N	8	18	97	121	74	68.47	152.346	0.000*
	%	2.5%	5.7%	30.5%	38.1%	23.3%			
Having to cope with stress.	N	9	20	84	127	78	69.26	149.767	0.000*
	%	2.8%	6.3%	26.4%	39.9%	24.5%			
Bounce back after hardships.	N	8	24	98	120	68	66.98	142.189	0.000*
	%	2.5%	7.5%	30.8%	37.7%	21.4%			
Believe that most things happen for a reason.	N	2	14	82	106	114	74.84	171.874	0.000*
	%	0.6%	4.4%	25.8%	33.3%	35.8%			
Give my best effort.	N	2	9	60	108	139	79.32	227.126	0.000*
	%	0.6%	2.8%	18.9%	34.0%	43.7%			
Believe I can achieve my goals.	N	1	15	64	113	125	77.20	196.403	0.000*
	%	0.3%	4.7%	20.1%	35.5%	39.3%			
Do not give up.	N	5	11	79	114	109	74.45	173.572	0.000*
	%	1.6%	3.5%	24.8%	35.8%	34.3%			
Know where to turn for help.	N	7	18	87	122	84	70.28	151.843	0.000*
	%	2.2%	5.7%	27.4%	38.4%	26.4%			
Stay focused and think clearly.	N	6	17	96	132	67	68.63	176.560	0.000*
	%	1.9%	5.3%	30.2%	41.5%	21.1%			
Prefer to take the lead in solving problems.	N	4	18	85	136	75	70.44	180.208	0.000*
	%	1.3%	5.7%	26.7%	42.8%	23.6%			
Not easily discouraged by failure.	N	7	18	88	123	82	70.05	153.226	0.000*
	%	2.2%	5.7%	27.7%	38.7%	25.8%			
Dealing with life’s challenges.	N	2	13	74	120	109	75.24	184.044	0.000*
	%	.6%	4.1%	23.3%	37.7%	34.3%			
Make unpopular or difficult decisions.	N	19	32	125	94	48	59.43	124.610	0.000*
	%	6.0%	10.1%	39.3%	29.6%	15.1%			
Able to handle unpleasant or painful feelings.	N	4	18	99	123	74	69.26	165.428	0.000*
	%	1.3%	5.7%	31.1%	38.7%	23.3%			
Dealing with life’s problems.	N	13	24	118	118	45	62.42	163.415	0.000*
	%	4.1%	7.5%	37.1%	37.1%	14.2%			
having a strong sense of purpose in life.	N	2	8	67	115	126	77.91	211.214	0.000*
	%	0.6%	2.5%	21.1%	36.2%	39.6%			
Feel in control of your life.	N	5	14	82	119	98	72.88	164.862	0.000*
	%	1.6%	4.4%	25.8%	37.4%	30.8%			
I like challenges.	N	11	22	81	123	81	68.95	135.711	0.000*
	%	3.5%	6.9%	25.5%	38.7%	25.5%			
Work to attain my goals.	N	8	12	74	127	97	73.03	172.912	0.000*
	%	2.5%	3.8%	23.3%	39.9%	30.5%			
Pride of my achievements.	N	8	10	79	90	131	75.63	179.893	0.000*
	%	2.5%	3.1%	24.8%	28.3%	41.2%			

Table 4 shows the resilience level, where the score for resilience was an average of 162 with 50.9% from the participants, followed by a high (136, 42.8%) and weak (20, 6.3%). The range of results was 23-100 with a mean ± SD of 71.164 ± 15.230. 

**Table 4:** Level of resilience in the study sample (n = 318). CD-RISC-25: Connor-Davidson Resilience Scale 25

CD-RISC-25	N	%	Score	
Weak	20	6.3	Range	Mean ± SD
Average	162	50.9	23-100	71.164±15.230
High	136	42.8		
Total	318	100.0		

Table 5 shows the mean and standard deviation calculated for the anticipated turnover items in the study sample (n = 318) using a one-sample *t*-test. The results found that the highest item was “If I got another job offer tomorrow, I would give it serious consideration” (Item 5) (mean ± SD 5.110 ± 1.719). Meanwhile, the lowest item was “I plan to stay in my job" (Item 1) (mean ± SD 2.991 ± 1.682). All items in this result show a statistically significant relationship to the intention to leave, with a *p*-value < 0.05, except item 6, i.e., “I have no intentions of leaving my current position,” with a *p*-value of 0.099>0.05.

**Table 5:** Mean and standard deviation calculated for the anticipated turnover items in the study sample (n = 318) using a one-sample t-test. The *p*-value has been calculated using a one-sample *t*-test. *Significant at the *p *< 0.05 level.

Items	Anticipated turnover among the nursing staff			One-sample T-test value (3.5)	
	Mean	±	SD	t	P-value
I plan to stay in my job.	2.991	±	1.682	-5.401	0.000*
I am quite sure that I will be leaving my job in the near future.	4.918	±	1.749	14.459	0.000*
Deciding to stay or leave my position is not an essential issue for me at this point.	3.242	±	1.787	-2.573	0.011*
I know whether I'll be leaving this agency shortly.	4.695	±	1.709	12.472	0.000*
If I got another job offer tomorrow, I would give it serious consideration.	5.110	±	1.719	16.699	0.000*
I have no intentions of leaving my current position.	3.336	±	1.765	-1.653	0.099
I have been in my job for as long as I want to.	4.934	±	1.534	16.673	0.000*
I am sure I will be staying here.	3.057	±	1.537	-5.144	0.000*
I do not have any specific idea how much longer I will stay.	3.075	±	1.664	-4.548	0.000*
I plan to hang on to this job.	3.151	±	1.632	-3.813	0.000*
There are big doubts in my mind as to whether or not I will stay in this organization.	4.230	±	1.715	7.586	0.000*
I plan to leave this position shortly.	4.142	±	1.796	6.369	0.000*

Table 6 shows nurses' intention to leave, with an average intention expressed by 233 (73.3%), followed by weak (73, 23.0%) and high (12, 3.8%). The range of results was 21-78 with a mean ± SD of 46.881 ± 8.732. 

**Table 6:** Level of nurses' intention to leave in the study sample (n = 318).

Anticipated turnover among the nursing staff	N	%	Score	
Weak	73	23.0	Range	Mean ± SD
Average	233	73.3	21-78	46.881 ± 8.732
High	12	3.8		
Total	318	100.0		

Table 7 and Table 8 show the relationship distribution between resilience and sociodemographic data and between resilience and working characteristics. The study concluded that no significant relationship existed between resilience and sociodemographic data, including age, gender, marital status, number of children, nationality, education level, years of experience as a nurse, working hospital, and monthly salary, and between resilience and working characteristics, including working unit/department, working shift, and working time (hours/day). Overall, the study sample did not significantly correlate with resilience.

**Table 7:** Statistical differences in the total mean nurses' resilience score in relation to demographic data. The *p*-value was calculated using an analysis of variance (ANOVA) or a *t*-test. * Significant at the *p *< 0.05 level.

Demographic data		N	CD-RISC-25			T or F	ANOVA or T-test	
			Mean	±	SD		Test value	P-value
Sex	Female	277	71.101	±	14.910	T	-0.190	0.850
	Male	41	71.585	±	17.440			
Nationality	Saudi	103	71.835	±	16.305	T	0.524	0.601
	Non-Saudi	215	70.842	±	14.716			
Age	<30 years	110	70.018	±	17.478	F	1.915	0.127
	30-40years	150	72.933	±	13.987			
	40-50years	49	67.673	±	13.616			
	≥50 years	9	74.667	±	10.392			
Marital status	Married	182	71.626	±	14.278	F	0.868	0.458
	Single	122	69.959	±	16.428			
	Widowed	4	71.250	±	22.897			
	Divorced	10	77.400	±	14.144			
Number of children	No children	171	69.807	±	16.395	F	1.296	0.276
	1 child	51	73.725	±	12.303			
	2 children	57	73.228	±	15.269			
	3 and more children	39	70.744	±	12.888			
Education	Diploma	49	69.041	±	17.057	F	1.228	0.300
	Bachelor's degree	241	71.253	±	14.997			
	Master's degree	24	75.625	±	13.217			
	Doctoral degree	4	65.000	±	15.427			
Working hospital	King Fahad Hospital	117	69.795	±	14.850	F	0.963	0.383
	Prince Mashari Hospital	92	72.728	±	16.291			
	Al-Mikhwah general Hospital	109	71.312	±	14.705			
Years of experience at the current hospital	<1 year	38	67.553	±	19.987	F	1.637	0.181
	1-5 years	132	70.917	±	14.256			
	5-10 years	73	70.575	±	14.835			
	>10 years	75	74.000	±	14.308			
Monthly allowance	<10 SR	174	71.057	±	15.561	F	1.332	0.265
	10-20 SR	95	69.811	±	15.391			
	>20 SR	49	74.163	±	13.508			

**Table 8:** Statistical differences in the total mean nurses' resilience score to working characteristics. The *p*-value was calculated using an analysis of variance (ANOVA) or a *t*-test. * Significant at the *p *< 0.05 level.

Demographic data		N	CD-RISC-25			T or F	ANOVA or T-test	
			Mean	±	SD		Test value	P-value
Working departments	Critical care units	33	74.455	±	13.700	F	0.642	0.721
	Surgical departments	38	69.605	±	14.346			
	Obstetrics and gynecology	29	70.690	±	14.575			
	Emergency department	37	71.459	±	15.996			
	Medical departments	33	68.061	±	17.904			
	Outpatients	51	70.608	±	15.121			
	Pediatric departments	41	70.415	±	14.066			
	Other	56	73.214	±	16.009			
Working shifts	Day	119	72.429	±	15.529	F	0.807	0.447
	Evening	13	68.154	±	18.434			
	Rotating day, evening, and night	186	70.565	±	14.818			
Working time (hours/day)	Eight hours	130	72.438	±	16.211	F	0.902	0.407
	Ten hours	43	69.233	±	12.085			
	Twelve hours	145	70.593	±	15.156			

Table 9 and Table 10 show the relationship distribution between nurses’ intentions to leave and sociodemographic data and between nurses’ intentions to leave and working characteristics. The findings of this study revealed significant associations between turnover intention and demographic variables, such as nationality, marital status, number of children, education level, years of experience at the current hospital, monthly allowance, working departments, working shifts, and working time (hours per day). The other results reflected no significant difference between intention to leave and age, with a *p*-value of 0.594 and *F* = 0.634. In addition, the ANOVA test results revealed no considerable difference between nurses’ intentions to leave and working hospitals (*p*-value = 0.539, *F* = 0.619); regarding gender, there is no significant relation between nurses’ intention to leave and gender (*p*-value = 0.301, T = 1.037).

**Table 9:** Statistical differences in the total mean nurses' intention to leave score in relation to the sociodemographic characteristics. The *p*-value was calculated using an analysis of variance (ANOVA) or a *t*-test. * Significant at the *p *< 0.05 level.

Demographic data		N	Anticipated turnover among the nursing staff			T or F	ANOVA or T-test	
			Mean	±	SD	T	Test value	P-value
Sex	Female	277	47.076	±	9.006		1.037	0.301
	Male	41	45.561	±	6.531			
Nationality	Saudi	103	43.757	±	8.979	T	-4.550	<0.001*
	Non-Saudi	215	48.377	±	8.221			
Age	<30 years	110	46.482	±	8.125	F	0.634	0.594
	30-40years	150	47.467	±	9.381			
	40-50years	49	46.490	±	8.029			
	≥50 years	9	44.111	±	8.838			
Marital status	Married	182	46.824	±	8.801	F	3.240	0.022*
	Single	122	47.738	±	7.662			
	Widowed	4	40.000	±	20.050			
	Divorced	10	40.200	±	11.144			
Number of children	No children	171	47.450	±	7.897	F	4.607	0.004*
	1 child	51	47.667	±	11.217			
	2 children	57	47.737	±	7.830			
	3 and more children	39	42.103	±	8.623			
Education	Diploma	49	43.551	±	9.120	F	3.109	0.027*
	Bachelor's degree	241	47.631	±	8.778			
	Master's degree	24	46.042	±	6.450			
	Doctoral degree	4	47.500	±	3.317			
Working hospital	King Fahad Hospital	117	47.419	±	7.964	F	0.619	0.539
	Prince Mashari Hospital	92	46.076	±	6.857			
	Al-Mikhwah general Hospital	109	46.982	±	10.733			
Years of experience at the current hospital	<1 year	38	45.421	±	8.532	F	2.747	0.043*
	1-5 years	132	48.038	±	7.881			
	5-10 years	73	47.658	±	9.217			
	>10 years	75	44.827	±	9.449			
Monthly allowance	<10 SR	174	47.724	±	8.104	F	7.462	0.001*
	10-20 SR	95	44.147	±	8.693			
	>20 SR	49	49.184	±	9.804			

**Table 10:** Statistical differences in the total mean nurses' intention to leave score to working characteristics. The *p*-value was calculated using an analysis of variance (ANOVA) or a *t*-test. * Significant at the *p *< 0.05 level.

Demographic data		N	Anticipated turnover among the nursing staff			T or F	ANOVA or T-test	
			Mean	±	SD		Test value	P-value
Working departments	Critical care units	33	46.606	±	7.802	F	2.949	0.005*
	Surgical departments	38	49.579	±	8.242			
	Obstetrics and gynecology	29	47.069	±	7.531			
	Emergency department	37	50.838	±	9.488			
	Medical departments	33	46.818	±	8.141			
	Outpatients	51	43.824	±	10.684			
	Pediatric departments	41	44.829	±	7.867			
	Other	56	46.821	±	7.469			
Working shifts	Day	119	44.891	±	9.621	F	5.268	0.006*
	Evening	13	46.615	±	7.911			
	Rotating day, evening, and night	186	48.172	±	7.958			
Working time (hours/day)	Eight hours	130	44.677	±	8.776	F	7.784	0.001*
	Ten hours	43	47.279	±	6.386			
	Twelve hours	145	48.738	±	8.891			

**Figure 1:**
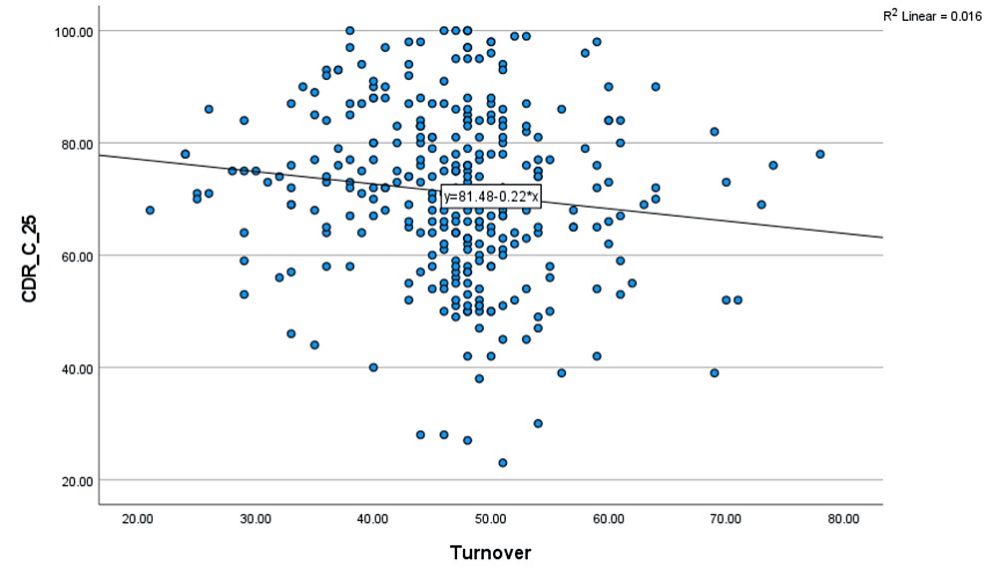
A Pearson correlation test was used to calculate the correlation between nurses' intention to leave and resilience. The *p*-value has been calculated as significant at the *p *< 0.05 level.

Figure 1 shows a significant negative, weak relationship between nurses’ intention to leave and resilience (r = -0.126, *p*-value = 0.024). Ultimately, the correlation was a significant negative relationship between nurses’ intention to leave and resilience. The negative relationship explains a low nurse’s intention to leave if there is a high level of resilience.

## Discussion

Resilience

The level score for resilience was moderate for most participants, with 162 participants having a moderate level of resilience (50.9%), followed by high (136, 42.8%) and weak (20, 6.3%). However, the scores ranged from 23 to 100, with a mean ± SD of 71.164 ± 15.230. This indicates that the participants already used protective factors, even unknowingly, to cope with adverse work conditions. These results are consistent with those of Alameddine et al. [9], who evaluated the resilience of the nursing workforce and factors related to the resilience of nurses employed by Lebanon’s primary COVID-19 referral center using data from 265 nurses. The findings suggest that nursing stakeholders at the system and organizational levels must work together to assess and enhance nurses’ resilience. The resilience displayed by the participants was moderate. Furthermore, the results of this study are consistent with Almegewly et al. [13], who conducted a cross-sectional correlational survey of critical care nurses in Riyadh City to assess perceived stress and resilience levels among critical care nurses. The investigator employed the CD-RISC-10, finding that most critical care nurses reported moderate resilience. In contrast to the variance in the findings of this study, according to Bozda and Ergün’s study conducted in Turkey [14], healthcare workers have higher levels of psychological resilience in their later years, with doctors having the lowest levels of this category. By contrast, Lin et al. [15] sought to evaluate the resilience of foreign medical professionals sent to assist local medical professionals in managing the 2019 new coronavirus disease (COVID-19) outbreak in China. Their findings revealed a significant degree of resilience among healthcare personnel. The findings underscore the significance of individual resilience in reducing the impact of burnout and employee attrition, particularly during periods of crisis. Possible reasons for the various research findings could be differences in the study period, study participants, and design and the multiple cultures involved in the investigations.

The relationship between resilience and demographic data (e.g., age, gender, marital status, number of children, nationality, education level, years of experience as a nurse, working hospital, unit/department, working shift, monthly salary, and working time (hours/day)) was investigated. Overall, the study sample did not significantly correlate with resilience, consistent with the findings of another cross-sectional survey by Alameddine et al. [9] in Lebanon, who discovered there was no discernible variation in participants’ resilience according to their age, marital status, degree of education, and experience. In addition, a cross-sectional study in Saudi Arabia by Alharbi et al. [16] suggested that work shifts, education level, and nationality were all significant factors in resilience among Saudi critical care nurses. By contrast, the factors of age and sex were not significant. Moreover, the results do not agree with the studies that found an association between resilience and other characteristics. Researchers have reported that resilience is affected by certain factors, mainly education and work experience, adding them to factors, such as age, gender, marital status, and working hours associated with resilience. Ang et al. [17] suggested that resilience substantially correlates with age group, work grade, years of nursing experience, education level, and marital status. Marzo et al. [18] found that income affects resilience. Furthermore, Oksuz et al. [19] found a statistically significant relationship among resilience and age, gender, work experience, and working hours. The differences in the findings can be attributed to the setting of the studies.

Nurses' intention to leave

By calculating the standard deviation and arithmetic means of each of the ATS items in the study sample, the level of staff nurses’ intent to leave was determined. The level of nurses’ intention to leave showed that most of the participants averaged 233 (73.3%), followed by 73 (23.0%), but the high was 12 (3.8%), whereas the results ranged from 21 to 78, with a mean ± SD of 46.881 ± 8.732. These results align with several other research studies in which nurses participated with a moderate intention to leave. In their cross-sectional design to investigate the factors influencing Saudi Arabian nurses’ intention to leave their current positions, Albougami et al. [1] discovered that nurses’ intention to leave was moderate. By contrast, in several studies, the intention to leave was higher than that in the current study. Ayalew and Workineh [20] carried out a cross-sectional study in Ethiopia to evaluate nurses’ intentions to quit, and related factors revealed nurses’ intentions were high. The results highlighted the importance of hospital and health center managers maintaining recognition at work to retain nurses. One possible reason for these variations between the study results was the different work experiences, workloads, and healthcare facility work environments, which could affect nurses’ intentions to leave.

In addition, there was a correlation between the intention of nurses to leave and the following demographic information: marital status, number of children, nationality, education level, years of nursing experience, unit, working shift, monthly compensation, and hours worked per day. With a T value of -4.550 and a *p*-value of 0.001, non-Saudi nurses were likelier than Saudi nurses to declare a desire to leave, in line with Albougami et al. [1]. There was a higher likelihood of turnover intentions among nurses who have never married, with a *p*-value of 0.022 and an *F*-value of 3.240. This finding is consistent with Albougami et al. [1]. One possible reason for this might be that nurses who have never married may have less skill in adapting to tricky situations and fewer family responsibilities so they can find more opportunities. Regarding the number of children, a significant relationship was found relating to nurses’ intentions to leave; nurses with three or more children were likelier to stay than others. This was supported by earlier research on 508 PHC nurses in the Jazan Region of Saudi Arabia by Almalki et al. [21], who proposed that respondents with children were less likely than those without children to declare a desire to depart. With a *p*-value of 0.027 and an *F*-value of 3.109, the current study also discovered a significant correlation between the educational level of nurses and their intention to leave, in line with Almalki et al. [21]. Years of experience significantly predicted the desire to leave, with a *p*-value of 0.043 and an *F*-value of 2.747. However, Maleki et al. [22] did not discover any statistically significant results.

Regarding the study’s results, Hoseini et al. [23] noted that nurses’ intentions to quit and their shift work were significantly correlated, with morning-shift workers having lower intentions to leave. This contrasts with Maleki et al. [22], who discovered no connection between the type of shift and nurses who intended to quit. Moreover, work units could influence turnover intentions (*p*-value = 0.005, *F* = 2.949). Inpatients, emergency rooms, surgical and medical units, critical care units, and obstetrics and gynecology units have higher turnover intentions than nurses in outpatient units. This is parallel to Qowi et al. [24] and may be related to the stress level and workload the nurses face in the patient area, increasing their stress and leading to a high intent to leave.

The nurses’ intention to leave was substantially correlated with their working hours, with *p*-value = 0.001 and *F* = 7.784, and the intention to leave increased for nurses who worked 12 hours. This was supported by a study conducted in the United States by Bae [25], who found that longer weekly work hours increased nurse turnover, as turnover increased when nurses worked ≥12 hours. The salary level has a substantial correlation with the intention to leave (*p*-value = 0.001, *F* = 7.462). The study’s findings demonstrated that high-salary participants firmly intend to leave. This finding is consistent with a study in Ethiopia by Worku et al. [26], who found that, compared to healthcare professionals whose family income was high, those whose monthly income was lower were less likely to plan to leave their current workplace. As a result, individuals with higher monthly incomes for families may be more likely to move because living in cities will improve their quality of life. However, the results of the current study, where the *p*-value was greater than 0.05 (significant level), indicate no significant relationship exists among gender, age, working hospital, and intention to leave. This is consistent with a study conducted in Iran by Maleki et al. [22]; the research also revealed no statistically significant correlation among age, gender, and intention to leave.

Relationship between resilience and nurses’ intention to leave

Figure 1 shows a negative relationship between the variables, where all *p*-values are less than 0.05 and all correlation coefficients are negative, to determine how resilience and nurses’ intention to quit are related. If there was a great degree of resilience, the negative connection would explain why there would be little intent to leave. Sellers et al. [27] noted that resilience is the only other factor that predicts a nurse’s intention to leave the unit, with more resilient nurses reporting a lower intention to leave, which reinforces the idea. Moreover, Shaabna et al. [28] highlighted the importance of high resilience levels in reducing future turnover intentions. Shaabna et al. [28] emphasized that nursing students’ resilience may be necessary for alleviating global nurse shortages by improving academic accomplishment and decreasing future intentions to leave and reducing burnout. Despite the previous findings, several research studies have failed to reveal a significant relationship between higher levels of resilience and the intention to leave. Two studies support this finding: Bernard [29] conducted a quantitative, nonexperimental, correlational design study in the United States to ascertain the connections between resilience, job satisfaction, and expected turnover among CNOs and found that resilience and intention to leave were not significantly correlated. Zhao et al. [30] conducted a cross-sectional descriptive survey in Changchun with 350 participants to investigate the mediating impact of job satisfaction and social support on turnover intention and resilience. Through social support and satisfaction with work, the researchers discovered that resilience indirectly affects the intention to leave a position.

Limitations of the study

The suggested study's limitations are associated with its methodological processes. The study’s restriction resulted from its exclusive focus on three organizations in southern Saudi Arabia. Consequently, it will not be generalizable to every healthcare facility in Saudi Arabia. Future research must expand the sampling scope and repeat it in various hospitals and organizations to determine whether the same outcomes would occur and examine additional possible variables affecting nurses' resilience and intention to leave. Second, this study’s cross-sectional design means causal linkages are not meant to be recognized. Further research must be conducted for a longitudinal study to investigate cause and effect.

Recommendations

This study has several implications for improving resilience and reducing the intention to leave among healthcare workers, especially nurses: First, hospital management must consider the amount of work and the excessive work schedule by assigning tasks to employees, minimizing their workload through flexible work schedules, shorter duty hours, and adequate breaks to lower work-related stress levels, and fostering teamwork among coworkers to reduce nurses’ intentions to leave. Second, creating orientation programs for newly hired nurses improves resilience and reduces the risk of employee turnover. In addition, future studies should take part in qualitative and longitudinal studies. The findings would be repeated in various hospitals and organizations to determine whether the same outcomes would occur. Lastly, a comparable study involving different groups of healthcare workers and experts could be carried out to determine whether the research’s conclusions differ significantly.

## Conclusions

This investigation produced several significant conclusions: the capacity to measure personal resilience, ascertain the degree of the intention of leaving among staff nurses, and determine the relationship between staff nurses' intention to leave and resilience. The findings show a weak but statistically significant negative correlation between nurses' intention to leave and resilience. There was no significant relationship between sociodemographic characteristics and resilience. Moreover, the results reveal a significant correlation between the intention of nurses to leave a hospital, nationality, marital status, number of children, education level, years of experience at the current hospital, monthly allowance, working departments, working shifts, and working time (hours per day). Thus, developing a strategy of intervention programs to assist nurses in adapting and building their resilience to reduce their intention to quit their jobs necessitates the leadership of the organizational administrator.
